# The Food Systems in the Era of the Coronavirus (COVID-19) Pandemic Crisis

**DOI:** 10.3390/foods9040523

**Published:** 2020-04-22

**Authors:** Charis M. Galanakis

**Affiliations:** 1 Research & Innovation Department, Galanakis Laboratories, 73131 Chania, Greece; cgalanakis@chemlab.gr; 2 College of Science, King Saud University, Riyadh 11451, Saudi Arabia; 3 Food Waste Recovery Group, ISEKI Food Association, 1190 Vienna, Austria

**Keywords:** COVID-19, vitamins, food safety, food security, food waste, sustainability

## Abstract

The World Health Organization (WHO) declared the outbreak of coronavirus disease (COVID-19, broadly referred to as “*coronavirus*”) a global pandemic, while thousands of infections and deaths are reported daily. The current article explores the food systems in the era of the COVID-19 pandemic crisis. It provides insights about the properties of bioactive ingredients of foods and herbs for the support of the human immune system against infections before discussing the possibility of COVID-19 transmission through the food chain. It also highlights the global food security issues arising from the fact that one-third of the world’s population is on lockdown. Finally, it underlines the importance of sustainability in the food chain in order to avoid or reduce the frequency of relevant food and health crises in the future.

## 1. Introduction

Coronavirus disease (COVID-19, caused by the novel coronavirus SARS-CoV-2) is an easily transmissible disease that was identified within December 2019 and declared a pandemic by WHO on 11 March 2020 [1]. The first infections were linked (with some, but not firm evidence) to the Huanan Seafood Market (Wuhan, China) [2]. Zhou et al. [3] used sequencing technology to show that SARS-CoV-2 and bat coronavirus possess a similarity of gene sequence up to 96.2%, suggesting bats as the possible source of SARS-CoV2. As of 16 April 2020, over 2 million cases of COVID-19 and over 137,000 corresponding deaths have been reported in over 210 countries, where these numbers are growing exponentially daily [4,5]. The flu-like symptoms of COVID-19 usually appear 5–6 days after infection and include coughing, sore throat, fever, muscle and body aches [6], and even loss of smell or taste in some cases [7].

Since no cure or vaccine has been developed for COVID-19 disease yet, the scientific community and authorities are seeking knowledge and information for the short- and long-term management of the current and future pandemic crises, respectively. The food sector and its stakeholders are also in the spotlight, as food is necessary for human survival and cannot be lockdown. A severe pandemic causing more than a 25% reduction in labor availability could generate significant food shortages across the globe [8]. Authorities and research communities should quickly identify the most critical threats to the food system during a pandemic in order to implement mitigation measures.

**Figure 1:**
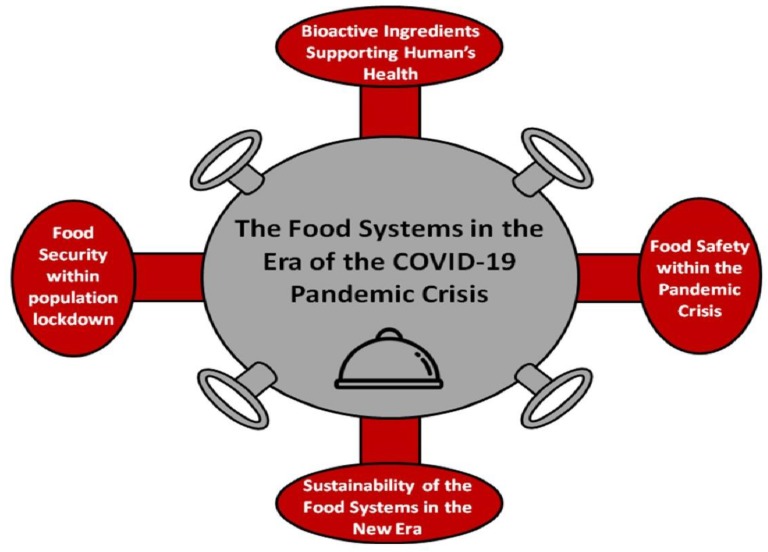
The food systems in the era of the coronavirus disease (COVID-19) pandemic crisis.

This article explores the food systems in the era of the COVID-19 pandemic crisis (Figure 1). There are four significant issues that the food industry and the food supply chain should address in the new era. Firstly, as consumers are looking to protect themselves and their immune system by adopting healthier diets, the availability of bioactive ingredients of food and functional foods may become critical, as the demand for these products may increase. Secondly, food safety is a significant issue in order to avoid the spreading of the virus between producers, retailers, and consumers. Thirdly, food security issues have emerged due to the lockdown of a billion people inside their houses. Last but not least, the sustainability of the food systems in the era of pandemics is another issue that the sector should address in order to restrict relevant crises in the future.

## 2. The Role of Bioactive Ingredients in Supporting the Human Immune System

The COVID-19 pandemic has similar characteristics to the well-known outbreaks of the last 20 years, such as the Severe Acute Respiratory Syndrome (SARS-CoV, from 2002 to 2004) and the Middle East Respiratory Syndrome (MERS, from 2012 until now) outbreaks. These two outbreaks were diseases of the lower respiratory tract with a similar clinical presentation at their early stages of infection (e.g., cough and fever), leading to significant mortality among vulnerable individuals (e.g., those who do not have a robust immune system and those who smoke) and older people [9].

The consumption of foods rich in vitamins and of functional foods can boost the immune system to help fight off viruses [10,11]. For instance, ascorbic acid (Vitamin C) is known to play a protective role, as it supports the immune function and is necessary for the development and repair of all body tissues [12]. Also, under certain conditions, it restricts the susceptibility of the lower respiratory tract to infection [13]. Foods rich in Vitamin C include citrus fruits, kiwifruits, and broccoli. Other vegetables, like carrots, spinach, and sweet potato, are rich in Vitamin A. This vitamin comprises a group of fat-soluble compounds (including retinol, retinoic acid, and β-carotene) that play an essential role in the immune function and are known to lower the susceptibility to infections [14]. For instance, isotretinoin (a derivative of vitamin A) mediates the down-regulation of angiotensin-converting enzyme 2 (ACE2), which is a crucial host cellular protein required for the entry of SARS-COV-2 in the body [15]. Besides, supplementation with Vitamins D and E may boost our resistance to COVID-19 [16], as the decrease in cattle’s levels of Vitamins D and E could lead to infection by a bovine coronavirus [17]. Das [9] suggested that the oral or intravenous administration of bioactive lipids (such as arachidonic acid and other unsaturated fatty acids) may aid in enhancing resistance and recovery from SARS-CoV-2, SARS, and MERS infections. Natural polyphenols such as hesperidin and rutin have been shown to be effective inhibitors of COVID-19 main protease (Mpro), which is considered a potential therapeutic drug target [18].

Herbal and Chinese medicines have also been shown to help in the treatment of viral diseases. For instance, ginseng root is useful in the prevention of viral respiratory diseases such as those due to strains of influenza [19,20]. *Astragulus membranaceus* is used to treat common cold and upper respiratory infections [21], whereas *Pelargonium sidoides* is an effective herbal remedy for the inhibition of respiratory viruses’ replication [20]. Historical evidence regarding the prevention of H1N1 and SARS influenza in the high-risk population indicates that Chinese herbal formulas could provide an alternative approach for the prevention of COVID-19 [21].

Other food bioactives found in traditional Chinese medicine (e.g., plant-derived phenolic compounds, flavonoids from litchi seeds, quercetin, and kaempferol) have been reported to inhibit the enzymatic activity of SARS 3-chymotrypsin-like protease (3CLpro). This enzyme is vital for the replication of SARS-CoV and thus could be suggested as a potential treatment agent against SARS-CoV-2 and supportive care agent for patients with COVID-19 [22]. The guidelines and the herbal formulas used for the supportive care of patients with COVID-19 were revised by Ang et al. [23]. However, the potential preventive effect of these formulas should be confirmed with rigorous and prospective clinical studies [21].

Dietary supplementation with the above vitamins, bioactive lipids, flavonoids, and herbs may be a tool to support the human immune system against COVID19. However, as of 16 April 2020, there is still no substantial evidence that these bioactive ingredients can boost enough our immune system to prevent or cure COVID-19. Nevertheless, their ability to boost the human immune system highlights their prospect use in functional foods and presence in nutraceuticals market. Nowadays, supporting the immune system is among consumers’ top health goals globally. In fact, almost one in five consumers listed immune system support as the number one reason for purchasing healthy products in a recent consumer survey (Nutraceuticalsworld, 2019; [24]). In the new era of the COVID-19 pandemic, it is foreseen that consumers will increasingly seek products to boost their immune system in the future.

## 3. Food Safety within the Pandemic Crisis

SARS-CoV, MERS, and SARS-CoV-2 may be traced to zoonotic transmission [9]. Coronaviruses circulate among animals, while some of them are also known to infect humans [25]. Although bats (as natural hosts) were a likely source of the initial SARS-CoV-2 infection, researchers and scientists are still seeking information and evidence of how COVID-19 is transmitted. Several other animals may also be relevant hosts, e.g., it is known that SARS-CoV-1 is transmitted to humans from civet cats, while MERS-CoV is transmitted to humans from camels [25]. According to the European Centre for Disease Prevention and Control (ECDC), the virus is spreading from person to person mainly via respiratory droplets that people cough, sneeze, or exhale [25]. The European Food Safety Authority (EFSA) and the United States Food and Drug Administration (FDA) are closely monitoring the transmission of COVID-19, which is affecting almost all countries around the globe, causing thousands of deaths. Previous outbreaks of related coronaviruses, particularly MERS-coronavirus (MERS-CoV) and SARS-coronavirus (SARS-CoV), showed that food is not a route of transmission for these relevant viruses [26,27]. At the moment (16 April 2020), there is no evidence to conclude that SARS-CoV-2 is different in this respect. Transmission is indeed possible if an infected individual touches food, and shortly afterward, another individual collects it and touches its eyes or mucous membranes of the mouth or throat [6,28]. Fresh foods may also be similarly exposed to SARS-CoV-2 before being frozen. In this case, the transmission may happen. For instance, it is known that MERS and SARS-CoV-1 can remain infectious for up to 2 years in a frozen state [28].

Thereby, the handling of packages should be followed by extensive hand washing or sanitizing in order to minimize any risk from touching food potentially exposed to coronavirus [29]. Besides, the FDA suggested that sanitization and cleaning of surfaces is a preferred precaution for food restaurants and kitchens compared to environmental testing for the COVID-19 virus [27]. Nevertheless, in some food-serving places, other precautions have been taken. For instance, some health authorities, restaurants, and cafeterias in Central Europe (Belgium) stopped serving rare steaks and meats [30]. However, these precautions are mainly related to food handling and preparation practices suggested by the WHO mainly to avoid cross-contamination between cooked and uncooked foods, including, as mentioned, cooking meat thoroughly and others (e.g., washing hands) [30].

## 4. Food Security with the Globe’s Population Lockdown

Since one-third of the world’s population is on lockdown (29 March 2020) [31], global food security alerts have arisen. Food systems incorporate all the different stages of food production from farm to fork (e.g., processing, distribution and preparation activities, consumption, and finally discharge) and various involved parts (e.g., infrastructure, agricultural inputs, landscape, farmers, retailers, shipping, and institutions) [32], and the lockdown has complicated the interactions among them.

According to Food and Agriculture Organization (FAO), supermarket shelves remained stock up until 29 March 2020. However, according to more recent reports, food supplies will be massively disrupted, and people going hungry could double, unless nations and governmental institutions act [33]. To avoid massive food shortages, it is of the highest importance that countries should keep the food supply chains going. In line with this, FAO is suggesting specific strategies, e.g., expanding emergency food assistance programs and providing immediate assistance to the agricultural production of smallholders by boosting e-commerce. Likewise, it proposes focusing on key logistics bottlenecks (e.g., hampered food transportation across provinces and perishable foods like fishery, vegetables, and fruits), addressing tax and trade policies to keep the supply chain moving and implementing fiscal measures in the case that food prices jump [34].

In the regions of Wuhan and Northern Italy, where COVID-19 prevailed in the first months of the pandemic, target measures by China and Italy, respectively, were taken banning profiteering, illicit trade, and hoarding of food products [35,36]. These measures restricted acute food shortages in the affected areas. Empty food shelves were observed temporarily (e.g., due to panic buying of supplies) and mainly in urban areas where vegetables like cauliflower and green onions could not be shipped out. Authorities approached food enterprises to gather information about their supplies of staple commodities like rice and fresh produce (e.g., vegetables) and connected them with sellers. Popular shopping mobile applications (apps) (e.g., the platform of Pinduoduo that assists farmers in finding alternative buyers in small city centers, e-commerce apps by JD.com and Alibaba Group) were also used for this purpose [36]. Besides, China assured the sufficient nourishment of the local population by releasing at least 300,000 tons of pork reserves [36]. Italy implemented relevant laws to force food makers to keep reserves for emergency purposes [35]. This practice resulted in a moderate reduction of agricultural production. However, according to industry sources, it is anticipated that prices will increase [35].

Other countries are also struggling to keep their food supplies available for the population. Indeed, in countries like U.S. and Japan, where governments do not often intervene in commerce, there are questions on how to balance the need to keep production going and the need to protect the workers [36]. Farmers are restricted in accessing markets, buying inputs, and selling products; thus, fresh produces remain at the farms and are lost or wasted. Intervention measures should target bringing smallholder producers closer to collection centers with capacity as well as facilitating e-commerce platforms to reduce mobility [34].

Besides, we have now an opportunity to promote practices and Industry 4.0 technologies suggested to tackle the well-known problem of food loss and food waste. In particular, information and communication technologies (ICTs), apps, Internet of Things (IoT) platforms, BIG Data, and artificial technology can be used to collect real-time data in order to improve the communication between suppliers and buyers and simplify the redistribution of foods. Apps using Big Data and artificial intelligence could be implemented to connect farmers and suppliers with markets and get acute responses if any alterations of demand occur [37]. Similarly, ICTs could be enacted during on-farm handling, postharvest, storage, and food transportation. For instance, the implementation of ICTs could extend the shelf-life of fresh produce by minimizing delays in the transport of imported products at the points of exit and entry [37]. ICTs can also help to monitor uncrewed vehicles and agricultural drones that have been suggested as a practice to reduce human contact in agriculture [34]. Value Stream Mapping is another tool that ensures the proper management of the supply chain from farm to fork and the identification of food loss-generating resources [38]. Several companies (e.g., IntechOpen Limited, Creately) have developed related software that offers the visualization of material flow and the monitoring of process steps for producing and delivering products.

The food supply system will also need to consider those items that are being stockpiled by replenishing them quickly and safely. The mobilization of food banks, non-governmental organizations, community-based groups, and private charities to deliver food (as families are staying home) during the lockdown period could help in this direction [34]. These institutions have relevant knowledge in managing delivery-at-home operations that are highly dependent on volunteer workers. Likewise, they have well-organized delivery routes and networks with farmers associations, retailers, and supermarkets. For example, food banks use apps (e.g., Food Cowboy) to collect unwanted surplus from food services and restaurants. The World Bank has developed an e-sourcebook to provide insights for agricultural smallholders and connect them with institutions and retailers [39]. Besides, food banks can also help countries, bringing collection centers of high capacity closer to smallholder producers in order to reduce the need for mobility [34].

## 5. Sustainability of Food Systems in the New Era of Pandemic Crises

Food systems affect human health directly and indirectly, and today it is more urgent than ever that they should become sustainable. In 2015, the United Nations (UN) 2030 Agenda for Sustainable Development declared the 17 Sustainable Development Goals (SDGs), which comprise an urgent call for action by developed and developing countries in a global partnership [40]. The food systems play a crucial role in achieving UN SDGs, e.g., to end hunger through achieving food security and improved nutrition (SDG2) and to ensure sustainable consumption and production (SDG12). Moreover, it is essential to halve the per capita global food waste at the retail and consumer levels, reduce food losses along supply chains (SDG 12.3), ensure healthy lives, and promote well-being for all ages [40]. For instance, the environmental and economic impacts of food waste make up at least 15% [41] of the impacts of the entire food value chain.

SDGs require the optimum utilization of all produced raw materials by the food systems and integrated activities throughout all stages of the food chain. Efforts begin by reducing postharvest losses [42] and then move on to processing and retailing, with the implementation of non-thermal technologies that ensure food safety [43,44,45,46,47], the recapture of bioactive compounds from food processing by-products, and their re-utilization in the food chain [48,49,50,51].

However, the current food systems are not sustainable. In particular, one-third (approximately 1.3 billion tn/year, which is equivalent to 3300 Mtn of CO_2_ emissions/year) of food produced globally is wasted [52]. A more recent estimate suggests that almost 14% of food is lost in stages before the retail level (e.g., agriculture, harvest, slaughter, and catch) [53]. As the global population is about to reach 9.8 billion by 2050 (UN, 2017), food security may decrease, leading to new food crises like the one of 2007–2008. Likewise, under these circumstances, people in territories of high population density seek food sources in all kinds of animals and insects. The safety and hygienic conditions of animal-based food sources during retailing and cooking in this kind of big local markets (often selling live animals) are challenging to be monitored by authorities. Cheng et al. [54] suggested that the possibility of the re-emergence of novel viruses from animals or laboratories should not be ignored, because bats comprise a vast reservoir of SARS-CoV-like viruses. Today, 13 years later, we are facing an outbreak of SARS-CoV2 that has common characteristics with bat coronavirus [3]. Likewise, the first infections occurred in the Huanan Seafood Market [2], where not only seafood, but also live and slaughtered bats, marmots, pheasants, snakes, deer, and organs of rabbits are sold [55]. Regardless of this being a coincidence or not, the world and subsequently the food systems should adapt in the new era of pandemics. There is evidence that the likelihood of pandemics has increased substantially over the past century due to urbanization, global travel, and integration, intensive exploitation of natural resources, and modifications in the use of land [56,57]. As urbanization and global population will increase over the next decades, this trend is expected to continue and intensify [39].

It is thus time to reconsider the food systems and design their future, i.e., it is essential to increase their resilience [58]. The current food systems are highly dependent on animal-based protein sources that are not sustainable from an environmental point of view but also form a health and food security perspective. For example, meat consumption is proportional to the amount of greenhouse gas (GHGs) emissions [59], whereas the consumption of red meat has been associated with chronic diseases like cancer [60]. Besides, the current food systems often have food safety gaps allowing the transmission of pathogenic microorganisms. The increasing demands for proteins, the increasing population, as well as the depletion of resources lead researchers to investigate more sustainable and safer food sources in order to feed the world and meet markets’ needs [61].

Nowadays, researchers are seeking alternative protein sources everywhere. For instance, cockroach milk and cockroach flour may play a pivotal role in the solution to the food shortage in the decades to come ([62,63]. The current trends in alternative protein sources include the dietary shift from beef to poultry and pork (to reduce red meat consumption) [64], new plant-based sources (e.g., quinoa), insects, microalgae [61], and artificial meat that is lab-grown under aseptic conditions. In some cases (e.g., the production of lab-grown meat), there is not enough evidence that the alternative protein sources under investigation are sustainable enough [65]. This status may change in the years to come if the production cost decreases. Besides, some of the alternative protein sources (e.g., microalgae) may also be a source of bioactive ingredients (e.g., polyphenols, flavonoids, lipids, and vitamins) that could be recovered and reutilized in the foreseen growing functional foods sector [61]. Food-processing by-products (from meat or fish processing or the dairy sector) comprise a rich source of proteins and other valuable compounds (e.g., antioxidants) that could be recovered and reintroduced in the food chain [42,46,61,66].

## 6. Conclusions

The COVID-19 pandemic crisis has created a new era. We are still trying to figure out the consequences for humanity, economy, and, subsequently, food systems. Academic researchers and food sector experts will have to face many significant challenges, e.g., ensuring food safety [67] and food security [68], introducing Industry 4.0 tools to reduce losses and waste of food, as well as identifying alternative and safe protein sources that meet the nutritional expectations of consumers [61]. At the same time, they should introduce innovations fast enough with the imminent economic crisis in the era of the COVID-19 pandemic, offering acceptable and economically competitive products and developing functional foods fortified with bioactive compounds and antioxidants that promote health and support consumers’ immune system. There is undoubtedly a need to avoid “business as usual” practices, to think out of the box and accelerate efforts to develop sustainable and modern food systems, e.g., to reduce the cost of aseptic lab-grown meat, reduce the cost of food waste recovery and reutilization in the food chain, and develop new and large food supply chains based on insects’ and microalgae proteins.
